# Diurnal variation in declarative memory and the involvement of SCOP in cognitive functions in nonhuman primates

**DOI:** 10.1186/s13041-023-01022-0

**Published:** 2023-03-25

**Authors:** Kimiko Shimizu, Ken-ichi Inoue, Takao Oishi, Masahiko Takada, Yoshitaka Fukada, Hiroo Imai

**Affiliations:** 1 Department of Biological Sciences, School of Science, The University of Tokyo, Tokyo, 113-0033 Japan; 2 Department of Pathological Cell Biology, Medical Research Institute, Tokyo Medical and Dental University, Tokyo, 113-8510 Japan; 3 Molecular Biology Section, Center for the Evolutionary Origins of Human Behavior, Kyoto University, Inuyama, Aichi 484-8506 Japan; 4 Systems Neuroscience Section, Center for the Evolutionary Origins of Human Behavior, Kyoto University, Inuyama, Aichi 484-8506 Japan; 5 Laboratory of Animal Resources, Graduate School of Medicine, Center for Disease Biology and Integrative Medicine, The University of Tokyo, Tokyo, 113-0033 Japan

**Keywords:** Diurnal variation, Nonhuman primates, Memory, Hippocampus, SCOP (PHLPP1)

## Abstract

Cognitive functions depend on the time of day in various organisms. Previously, we found that 24-h recognition memory performance of nocturnal mice changes diurnally through SCOP protein-dependent regulation. It remains unknown whether diurnal change and SCOP-dependent regulation of memory performance are conserved across species with diurnal/nocturnal habits. We tested whether the memory performance of diurnal Japanese macaques depends on the time of day. The memory association between bitter taste of drinking water and the nozzle color of the water bottle was established during the light period of the day to evaluate of memory performance for macaques. Here we found diurnal variation of declarative memory in Japanese macaques. The middle of the daytime is the most effective time for memory performance during the light period. To assess whether SCOP is involved in declarative memory performance, we interfered with SCOP expression by using lentiviral vector expressing shRNA against *Scop* in the hippocampus of Japanese macaques. *Scop* knockdown in the hippocampus abrogated the memory performance in the middle of the daytime. Our results implicate that SCOP in the hippocampus is necessary for the diurnal rhythm of the memory system and that the SCOP-dependent memory regulation system may be conserved in mammals.

**Supplementary Information:**

The online version contains supplementary material available at 10.1186/s13041-023-01022-0.

## Introduction

Daily rhythms are widely observed in a variety of physiological functions among many organisms. In mammals, blood pressure [1], body temperature [2], hormone secretion [3, 4], and even higher brain functions such as mood regulations [5] and memory performances [6] show the daily rhythms, which are controlled by the internal circadian clocks [5–7]. We previously found that nocturnal mice exhibited the highest performance of a recognition memory task during the early (subjective) night even in constant darkness [6]. Several studies have also demonstrated daily variations in spatial memory performance [8–10] and contextual/cued fear memory performance [7, 8, 11, 12] in rodents. In humans, on the other hand, the recognition memory task was performed better in the mid-afternoon than in the early morning [13, 14]. The hippocampus is known to contribute largely to the declarative memory formation in humans [15, 16], nonhuman primates [17], and rodents [6, 18–20]. It has been reported that the diurnal change in synaptic transmission in the hippocampus is maximal at night in nocturnal rats but during the daytime in diurnal monkeys [21]. Given that the integrity of the hippocampal formation is critical for cognitive behavior, nocturnal and diurnal animals possibly show maximal memory performances at different times of day.

SCOP (suprachiasmatic nucleus circadian oscillatory protein, later termed PHLPP1β [22, 23]) was initially identified as a protein whose expression is circadian-regulated in the rat SCN (suprachiasmatic nucleus) of the hypothalamus [24]. In rodents, the SCOP protein is predominantly expressed in the central nervous system, particularly enriched in pyramidal neurons in the hippocampal CA1 to CA3 [25] and other brain areas essential for memory formation [26, 27]. SCOP in the hippocampal pyramidal neurons is a critical mediator that links the memory formation with the circadian clockwork in mice [6]. Knockout or knockdown of *Scop* in the hippocampus caused a deficit in long-term recognition memory and blunted its circadian regulation in mice [6]. The amino acid sequences and the domains of SCOP were highly conserved across rodents and humans [24], implicating a common role in mammals.

In the present study, we addressed whether memory performance of a diurnal nonhuman primate, Japanese macaques, shows a time-of-day-dependent variation during the daytime (the light period) and whether SCOP regulates learning and memory performances. We established a behavioral test, the “color-taste association task,” to estimate the declarative memory performance of caged macaques. This task allows them to choose water bottles without severe aversion and, therefore, should be unrelated to a reward or fear response in the brain system. Because experiments under the constant light (or dark) condition are not allowed for nonhuman primates due to the regulations of research ethics, the monkeys were subjected to the memory task only during the 12-h light period. Here we found diurnal variations in declarative memory, indicating that SCOP plays an important role in memory performance.

## Results

### Diurnal variations in recognition memory

**Fig. 1:**
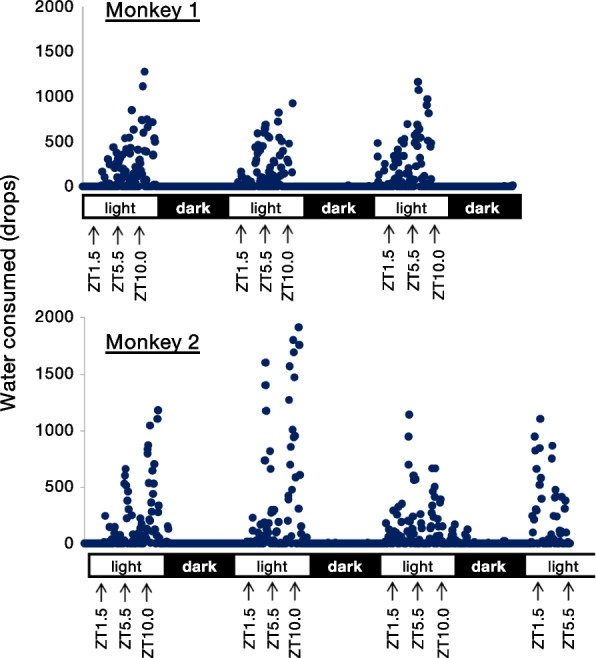
Daily water consumption from regular water supply system. Several days of water consumption of two macaques in 12-h light/dark condition. Blue dots are water drops the monkey consumed per 5 min. The monkeys are fed three times a day: ZT 1.5, ZT5.5, and ZT10.0 during a daytime

Before exploring diurnal variations in recognition memory by a color-taste association task utilizing water-drinking behavior in Japanese macaques, we monitored their daily water consumption from a regular water supply system in a 12-h light/dark cycle (Fig. 1). Two randomly-selected monkeys were fed with a diet three times a day: dawn (ZT [zeitgeber time] 1.5), midday (ZT5.5), and dusk (ZT10.0), where ZT0 is defined as light-on and ZT12 as light-off. We identified that these monkeys drank water mainly when fed. Therefore, we decided that the animals should be fed at the time of the task to let them motivate to drink water during the task.

**Fig. 2:**
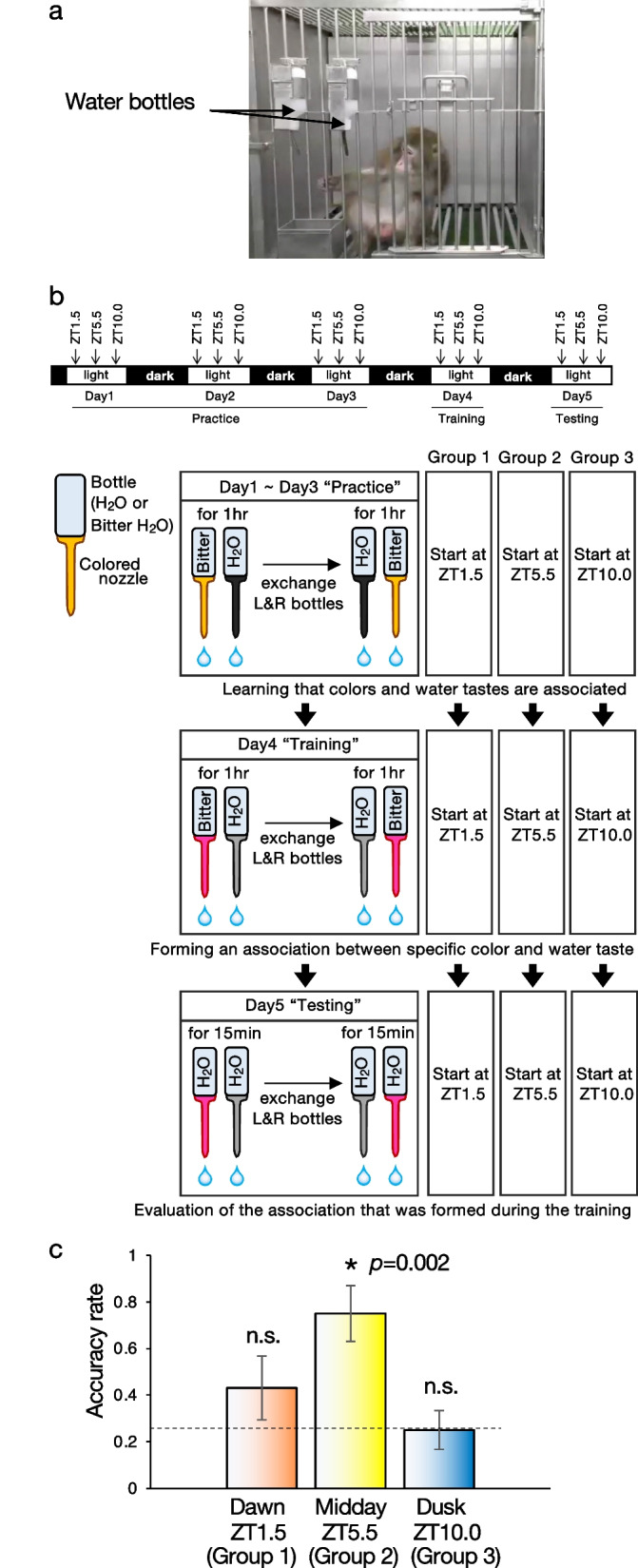
Diurnal change of color-taste association task. **a** A Japanese macaque in a cage with two water bottles. **b** The method for the color-taste association task. The color-taste association task is done in five consecutive days consisting of three parts, three days of “practice”, one day of “training”, and one day of “testing” at either three-time points, ZT 1.5 (Group 1), ZT 5.5 (Group 2), or ZT 10 (Group 3). In the “practice,” two bottles of bitter water and normal water equipped with nozzles in different colors were presented to the monkeys. The “practice” let animals learn that nozzle color is associated with water taste. Monkeys were allowed to freely drink water from the bottles for 2 h with the location exchange at 1-h after the bottle setting. “Training” and “testing” were carried out at the same time of day as the “practice,” and nozzle color sets are the same in the “training” and “testing” but different from those used in the “practice.” In the “training,” the bottles are presented for 2 h with the location exchange at 1-h after the bottle setting. In the “testing,” the two bottles were both filled with normal water, and the animals were allowed to drink water from the bottles for 30 min, with the location exchanged at 15-min after the bottle setting. An association between specific nozzle color and water taste formed during the "training” was evaluated in the” testing.” **c** The accuracy rate was examined at ZT1.5, ZT5.5, or ZT10. *p = 0.002 (ZT5.5) by Student’s *t*-test (versus 0.25 chance level). Error bars, s.e.m. (n = 6 macaques, a total of 16 task trials for ZT1.5, 13 task trials for ZT5.5, 16 task trials for ZT10.0). The dotted line represents performance by chance 0.25. n.s., not statistically significant

We explored diurnal variations in hippocampus-dependent recognition memory by examining monkey’s performance in the color-taste association task (Fig. 2a–c). Six monkeys reared in individual cages were subjected to the task at randomly-assigned either three-time points of the day: dawn (ZT 1.5), midday (ZT5.5), and dusk (ZT10). (Fig. 2a, b). The task was performed for five consecutive days consisting of three parts: 3-day “practice,” 1-day “training,” and 1-day “testing” (Fig. 2b). In the “practice,” two bottles of bitter water and normal water equipped with nozzles in different colors were presented to the monkeys to learn that colors and water tastes are associated. The animals were allowed to freely drink water from the bottles for 2 h with their position exchange 1 h after the bottle setting. The bottle position exchange eliminates an association between water taste and bottle position, left or right. “Training” and “testing” were performed by using a set of water bottles with nozzles having a color set different from that used in the “practice”. An association between specific nozzle color and water taste formed during the "training” was evaluated in the” testing”, during which the two bottles of the same color set as was used in the “training” were both filled with normal water. Then, the monkeys were allowed to freely drink water from the bottles for 30 min, during which the position of the bottles was exchanged 15 min after the bottle setting. In the “testing”, we monitored which color of a nozzle the animals had first selected 24 h after “training”, by which they had experienced an association between the bitter taste and the specific nozzle color. The memory of the association between the bitterness and the nozzle color during the “training” would let the animals drink water from the color nozzle that was not bitter in the “testing.” When the animals first chose the color nozzle with normal water both before and after the bottle exchange, then 1-point was given as it was judged that they remembered the association. This scoring method should exclude the case of selecting the right color nozzle by chance. The tasks were carried out 1–4 times for each time point for each monkey. The resulting points of the tasks (a total of 16 tasks for ZT1.5, 13 tasks for ZT5.5, and 16 tasks for ZT10.0 at each time point in six monkeys) were combined. The color-taste association task revealed an apparent diurnal change with the highest level at ZT5.5 midday among the three-time points during the light period (Fig. 2c). The accuracy rate at midday was significantly higher than the 0.25 chance level (dotted line). Since the color-taste association task determined midday as the best time of day for memory reinforcement, we then approached the molecular mechanism to augment declarative memory performance.

### Abolishment of color-taste association by *Scop* knockdown in hippocampus

**Fig. 3:**
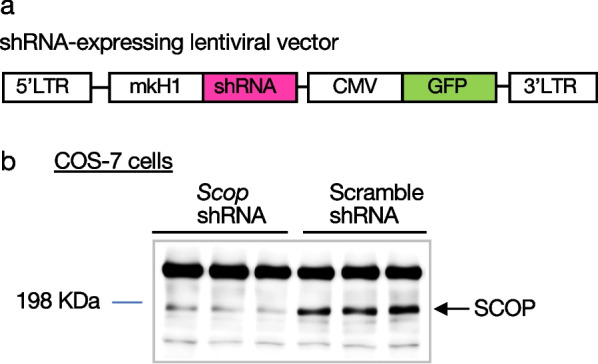
Evaluation for the knockdown efficiency of *Scop* shRNA expressing lentiviral vector. **a** Schematic diagram of the shRNA-expressing lentiviral vector. The shRNA is controlled by the macaque H1 promoter (mkH1), and the CMV promoter drives the EGFP marker gene for tracking transduced cells. 5′-LTR, HIV-1 5′-LTR; 3′ LTR, HIV-1 self-inactivating 3′-LTR. **b** Western blot analysis of SCOP protein level after shRNA expressing lentivirus infection. Decrease in SCOP protein level in COS7 cells by infection of anti-Scop shRNA lentivirus (sh*Scop*). Scramble shRNA lentivirus was used as a control. The sample in each lane is from a different culture dish. The displayed blot was cropped from the full-length blot in the Additional file 1

SCOP in the hippocampus is a critical molecule for diurnal regulation and memory reinforcement of recognition memory in nocturnal mice [6]. Of interest was to ask whether SCOP in the hippocampus might play a similar role in mice and diurnal mammalian species. To investigate the role of SCOP in the hippocampus in monkeys, we performed knockdown experiments of SCOP by using a lentiviral vector. The lentiviral vector was designed to express both *Scop* shRNA (short-hairpin RNA) driven by Japanese macaque H1 promoter (mkH1) and enhanced green fluorescent protein (GFP) driven by CMV promoter (Fig. 3a). The COS-7 monkey cell line was used to evaluate the knockdown efficiency of SCOP by the *Scop* shRNA lentiviral vector. Infection of the vector efficiently reduced the endogenous SCOP protein level as compared to COS-7 cells treated by the control Scramble shRNA lentiviral vector (Fig. 3b).

**Fig. 4:**
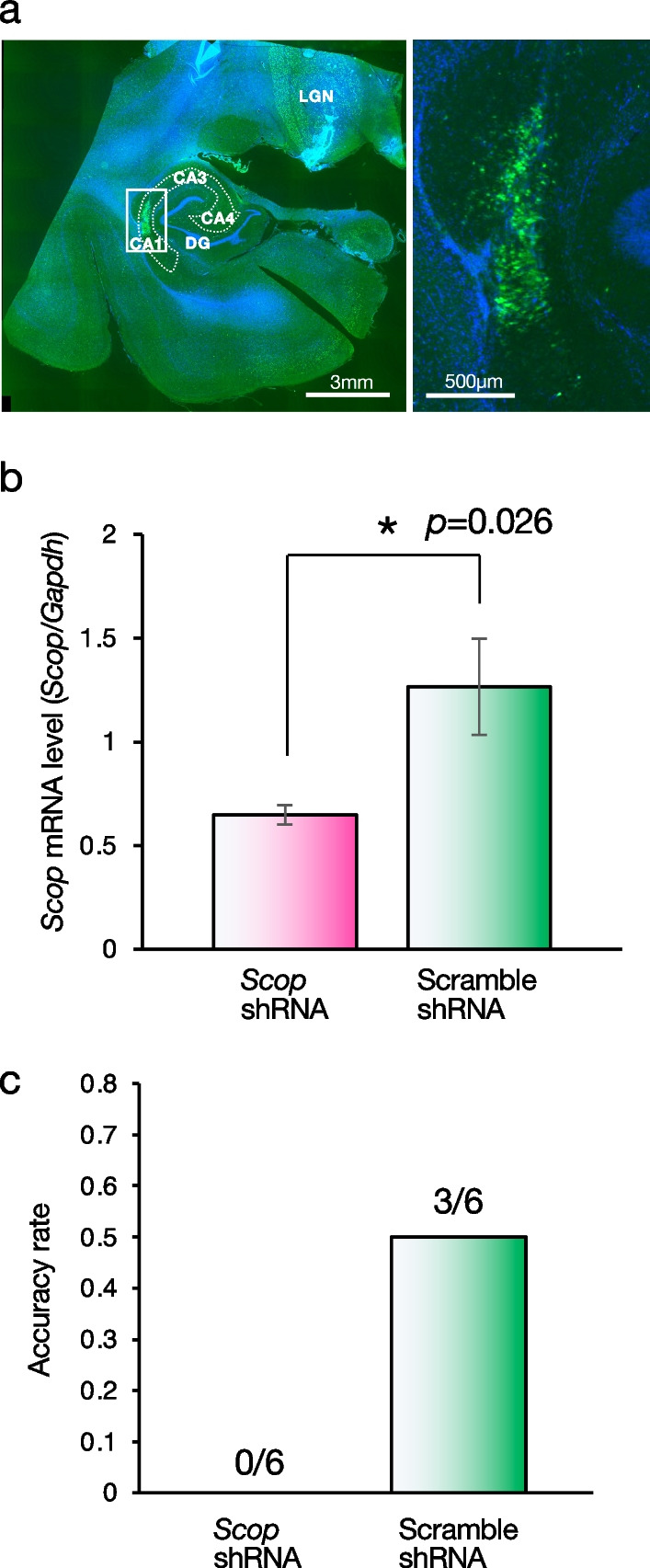
Effect of *Scop* knockdown in the hippocampus on color-taste association task. **a** A representative EGFP fluorescence (green) and Hoechst 33258 (Blue) image of a hippocampal section of the macaque that received shRNA lentivirus. The white square in the left photo is enlarged in the right photo. Scale bars are indicated in the photos. The white dotted area shows the hippocampal CA1 to CA4. DG, dentate gyrus; LGN, lateral geniculate nucleus. **b** Evaluation of the knockdown activity of anti-*Scop* shRNA lentivirus on *Scop* mRNA level. Decrease in *Scop* mRNA level in the hippocampus by infection of anti-*Scop* shRNA lentivirus (*Scop* shRNA). Scramble shRNA lentivirus was used as a control. Error bars, SEM (n = 6 subregions). **c** The accuracy rate for *Scop* knockdown and control macaques (one each) is shown. Memory performance at ZT 5.5 (midday) in each monkey that received lentivirus expressing *Scop* shRNA or Scramble shRNA were measured. The task was carried out six times for each animal. The number above each bar on the graph indicates the number of corrects for the number of trials

We randomly selected two monkeys, one for knockdown (*Scop* shRNA) and another for its control (Scramble shRNA). The shRNA lentiviral vectors were injected into the hippocampus. Fourteen injections were made into the bilateral CA1 regions (seven injections per unilateral CA1) of each monkey to cover the whole CA1 area positioned by an MRI-guided navigation system. After the behavioral analysis, the injection sites of the lentiviral vectors in the hippocampal CA1 region were confirmed by GFP immunostaining (Fig. 4a), indicating that the vectors were transfected at the targeted CA1 region. The efficiency of *Scop* knockdown was verified by quantitation of *Scop* mRNA in the hippocampus using RT-qPCR, and *Scop* mRNA in the hippocampus was reduced significantly to 51% in comparison with the control case delivered with Scramble shRNA (Fig. 4b).

After the lentiviral vector injections, the animals were allowed to recover for eight weeks, and then the color-taste association task was performed at ZT5.5 (midday), which was the highest performance time of day in the non-injected animals (Fig. 2c). Six trials of the color-taste association task were conducted over time for each animal. The *Scop* knockdown suppressed the performance of the color-taste association task, contrasting with the control animal that had been injected with the lentiviral vector expressing Scramble shRNA (Fig. 4c). The *Scop*-knocked-down monkey did not earn even 1-point over six trials (Fig. 4c *Scop* shRNA), whereas the control monkey correctly chose the colored nozzle before and after three over six trials (Fig. 4c Scramble shRNA). These results suggest that SCOP in the hippocampal CA1 is indispensable for the color-taste association memory in macaques, although our results from the SCOP knocked-down experiments are relatively premature, because of the small sample size. Further studies with a more sample size will confirm the conclusion.

## Discussion

The present study has established a color-taste association task to investigate memory performance using macaques reared inside cages. The memory performance of this task was the best at midday during the daytime (Fig. 2c). A similar result was reported on a human declarative memory task which was performed better in the mid-afternoon than in the early morning [13]. This similarity supports that the newly established color-taste association task is a reasonable method to verify memory ability. In our previous study, on the other hand, mice performed a recognition memory task better during the early night (their active phase) than during the resting phase [6]. Such a difference in the best timing for declarative memory performance would be dependent on the temporal habitat, nocturnal vs. diurnal. Synaptic excitability that is the highest at night in nocturnal rats but during the daytime in diurnal monkeys [21] may explain the difference in the best time of day for memory formation. On the other hand, the best timing for memory formation might depend on the task type; nocturnal mice perform a fear memory task better during a daytime (their inactive phase) [7, 11]. Considering these findings, we can assume that the diurnal regulation for learning and memory performance may be universal, although the timing of the best performance varies among animal species and types of tasks [28].

*Scop* is widely conserved in vertebrates [29, 30], implicating its crucial role throughout vertebrate evolution. The present results and our previous work [6] indicate that SCOP in the hippocampus has the ability to enhance memory capability and generate a diurnal rhythm of memory performance across species at an appropriate time of day. In mice, we revealed SCOP-dependent diurnal regulation of long-term memory through a mechanism that the SCOP levels in the hippocampal membrane rafts regulate the K-Ras-ERK-CREB pathway and consequently control the CRE-mediated transcription and long-term memory formation [6]. The SCOP protein level in the mouse hippocampal membrane rafts is higher during the active phase (nighttime), and, therefore, the amount of SCOP in the macaque hippocampal membrane rafts may be higher during the daytime (their active phase). Further investigations are needed to address this issue.

## Materials and methods

### Animals

Six adult Japanese macaques (*Macaca fuscata*, 7–10 kg, 5–10 years old) of either sex (four males and two females) were used in this study. The monkeys were housed in individual cages in a 12-h light/dark cycle. Animals were fed regularly with dietary pellets and had ad libitum access to water by a water supply system except for the experimental periods.

### Measurement of water consumption

Water consumption was measured by a drinkometer (O’Hara & Co., Tokyo, Japan) placed into a regular water supply system route when not conducting the memory task. The drinkometer records the number of drops consumed per 5 min.

### Color-taste association task

The color-taste association task was conducted with feeding/drinking limitations. In order to let the macaques more motivate the drinking behavior during the behavioral task, the regular water supply was stopped for 3 h before starting the behavioral paradigm for midday and dusk. For tests in the dawn, the regular water supply was stopped at a light-off time (ZT12) the day before the behavioral experiment. The behavioral tests were performed using a two-bottle system approved by the Animal Welfare and Animal Care Committee of the Primate Research Institute, Kyoto University (no. 2011-093). Briefly, two filled bottles (500 mL) were set in front of the monkeys: one contained bitter water containing 20 mM salicin (Sigma-Aldrich, MO, USA), and the other contained normal water in the “practice” and the “training” (see Fig. 2b). For the discrimination of the two bottles, stainless steel nozzles of the bottles were treated with oxidized coloration and stabilized in six different colorings, *i.e*., magenta, black, blue, brown, gray, and white (processed by Nakano Kagaku, Niigata, Japan).

The task was performed in five consecutive days consisting of three parts: three days of “practice,” 1-day “training,” and 1-day “testing.” In the “practice,” two bottles of bitter water and normal water equipped with nozzles in different colors were presented simultaneously to the macaques (see Fig. 2a). The three days of the “practice” let animals learn that nozzle color is associated with water taste. Monkeys were allowed to freely drink water from the bottles for 2 h with the location exchange at 1-h after the bottle setting. In the “training,” bitter water and normal water were presented to the same animals by bottles with nozzle color sets different from those used in the “practice.” Again, monkeys were allowed to freely drink water from the bottles for 2 h with the location exchange at 1-h after the bottle setting. An association between specific nozzle color and water taste formed during the "training” was evaluated in the” testing,” during which the two bottles of the same color set as was used in the “training” were both filled with normal water and presented at the same time. Then, animals were allowed to freely drink water from the bottles for 30 min, during which the location of the bottles was exchanged 15 min after the bottle setting. In this “testing” process, memory performance was assessed as the degree of correspondence between the nozzle color and water taste the animals had experienced in the “training.” All the “testing” process was recorded by a video camera. When the monkey in the “testing” first chose the nozzle color both before and after the bottle exchange that the animal had experienced normal water in it, then 1-point was given as it was judged that the animal remembered the association between the color and taste. The task (practice, training, and testing) was carried out a maximum of four times at each time point at ZT1.5, ZT5.5, or ZT10.0 (ZT10 means 10 h after the light ON) for each monkey. The total tasks at each time point in six monkeys were 16 for ZT1.5, 13 for ZT5.5, and 16 for ZT10.0. A different color set was used for each task trial at a single time points for each animal. The number of points divided by the trials was used as the accuracy rate. The inter-task interval was at least 1 month.

### Production of shRNA-expressing lentiviral vector

The plasmids, pENTR4-H1, CS-RfA-CG, pCMV-VSV-G-RSV-Rev and pCAG-HIVgp were provided by Dr. Hiroyuki Miyoshi, RIKEN Bioresource Center, Tsukuba, Japan. shRNA targeting *Scop* was designed using siDirect (http://design.RNAi.jp/), and the target sequence (GGATA TTGGC CATAA TCAAA CGTGT GCTGT CCGTT TGATT ATGGC CAATA TCCA) was used for the down-regulation of macaque *Scop*. A control shRNA with a scrambled sequence (GATAT GGCAC TGATA ATCAA CGTGT GCTGT CCGTT GATTA TCAGT GCCAT ATCA) was designed. The pairs of the complementary oligonucleotides containing these sequences were synthesized (SIGMA), annealed, and cloned into the modified pENTR4-H1, in which human H1promoter was replaced by Japanese macaque H1 promoter (mkH1; see Fig. 3a). Cloning of the mkH1 promotor was performed from the genome of a Japanese macaque with reference to the homologous region of the rhesus H1 sequence and the human H1 sequence at the UCSC genome browser (https://genome.ucsc.edu). The H1-shRNA fragment from pENTR4 H1-shRNA was then inserted into lentiviral vector CS-RfA-CG by the Gateway system (Invitrogen, CA, USA) to obtain CS-H1-shRNA-CMV-GFP.

HEK293T cells were transfected with transfer (CS-H1-shRNA-CMV-GFP), envelope, and packaging (pCMV-VSV-G-RSV-Rev and pCAG-HIVgp) plasmids by the polyethylenimine method. Eighteen hours after transfection, the medium was replaced with a fresh one, and after that, the cells were incubated for 24 h. Then, the medium was harvested and filtered through a 0.22 µm PVDF filter (Millipore, Burlington, MA, USA). The filtered medium of 32 ml was centrifuged with bottomed 20% (w/v) sucrose (5 ml) at 35,000 ×*g* for 2 h at 4 °C in a Beckman SW32 Ti. The pelleted viral particles were resuspended in 0.001% Pluronic-F68 in phosphate-buffered saline (PBS; pH 7.4) at 4 °C for 2–4 h.

For measuring RNA titer, viral RNA in 50 nL of the vector stock solution was isolated with a NucleoSpin RNA virus kit (Takara, Shiga, Japan), and the copy number of the RNA genome was determined by quantitative PCR using Taq-Man technology (Thermo Fisher Scientific, Waltham, MA, USA). The viral biological titers were also determined by infection of COS-7 cells with a dilution series and counting colonies of GFP-positive cells.

### Assessment for *Scop* knockdown lentiviral vector in vitro

To determine the efficiency of *Scop* shRNA expressing lentivirus, we chose monkey cell line COS-7. The COS-7 cells were infected with *Scop* shRNA or scrambled shRNA (Scramble shRNA) expression lentivirus (2 × 10^4^ ifu / 3.8 cm^2^ dish surface) and cultured for three days before western blot analysis for SCOP protein expression. For western blot analysis, proteins separated by SDS–PAGE were transferred to a polyvinylidene difluoride membrane (Millipore, USA). The blot was blocked in a blocking solution of 3% bovine serum albumin in T-TBS (0.05% Tween20, 50 mM Tris–HCl, 140 mM NaCl and 1 mM MgCl_2_; pH 7.4), for 2 h at room temperature. Then the blots were incubated for 4 h at room temperature with anti-SCOP antibody (1:2,000, αCB in Ref. 24) diluted in the blocking solution. The signals were visualized by an enhanced chemiluminescence detection system (PerkinElmer, Boston, MA, USA).

### Injections of lentiviral vectors

Based on the 3R principle (Replacement, Reduction, and Refinement) for animal experiments, lentivirus was administered to each macaque for *Scop* shRNA (one male) and Scramble shRNA (one female) in the experiment. The animals were first sedated with ketamine hydrochloride (5 mg/kg, i.m.) and xylazine hydrochloride (0.5 mg/kg, i.m.) and then anesthetized with sodium pentobarbital (20 mg/kg, i.v.). The monkeys were kept hydrated during the surgical operation with a lactated Ringer’s solution (i.v.). An antibiotic (Ceftazidime; 25 mg/kg, i.v.) and an analgesic (Meloxicam; 0.2 mg/kg, s.c.) were administered at the first anesthesia. After partial removal of the skull, multiple injections of each vector were performed into the hippocampal CA1 area with the aid of an MRI-guided navigation system (Brainsight Primate, Rogue Research, Montreal, QC, Canada). A total volume of 70 µL of each vector was injected into multiple sites (5 µL/site, seven sites per side, 14 sites per animal) through a 10 µL Hamilton microsyringe. The injection titer of the viral vector was 2 × 10^10^ gc/mL. After the injections were completed, the scalp incision was closed. All experiments were performed in a specific laboratory (biosafety level 2) established at the Primate Research Institute, Kyoto University, designed for in vivo animal infectious experiments.

### Immunohistochemical analysis

At the end of experiments in macaques, the animals were deeply anesthetized with sodium pentobarbital (50 mg/kg, i.p.) and perfused transcardially with PBS. The brains were cut in the coronal plane at the 3-mm thickness, and the slices containing the hippocampus were immersed in 4% paraformaldehyde in PBS overnight, followed by 30% sucrose in PBS for two days at 4° C and cut into serial coronal sections (20 µm) by a microtome in a cryostat (Leica, Germany). The sections were washed with 0.1% Triton-X100 in PBS for 15 min × 3 times and then blocked in 1% normal goat serum, 1% BSA, 0.1% Triton-100 in PBS for 1 h at room temperature, and incubated in anti-GFP antibody (1:1000, Invitrogen, G10362). The immunoreactivity was visualized with Alexa 488-conjugated goat anti-rabbit IgG (1:1000; Molecular Probes) and then stained with 1 ng/ml Hoechst 33342 (Sigma) to visualize nuclei. The hippocampal sections were imaged on BZ-9000TS Microscope (Keyence, Osaka, Japan).

### RT-q PCR analysis

Total RNA was isolated from six subregions of the macaque hippocampus using TRIzol reagent (Invitrogen) and was subsequently purified by RNeasy Mini kit (Qiagen) according to the manufacturer’s protocol. RT–qPCR analysis was performed using Go Taq 2-step RT–PCR system (Promega) in a Step One Plus (Applied Biosystems). Data are presented as values normalized to the housekeeping gene *Gapdh*. PCR primers used are;for *Scop* FW 5′-CCCCA GCTGT TTGGA GTCAT-3′ and RV 5′-TCAAA CACAC CGTAG AGGGC-3′ for *Gapdh* FW 5′-ACCGT GGTCA TGAGT CCTTC C-3′ and RV 5′-GCACC ACCAA CTGCT TAGCA-3′.

## Supplementary Information

**Additional file 1**: The full image of the western blot data shown in Fig.3b in the main manuscript. The blue square is the cropped area..

## Data Availability

The datasets generated and analyzed during the current study are available from the corresponding author on reasonable request.

## References

[1] Millar-Craig MW, Bishop CN, Raftery EB (1978). Circadian variation of blood-pressure. Lancet.

[2] Refinetti R, Menaker M (1992). The circadian rhythm of body temperature. Physiol Behav.

[3] Kaneko M, Hiroshige T, Shinsako J, Dallman MF (1980). Diurnal changes in amplification of hormone rhythms in the adrenocortical system. Am J Physiol.

[4] Oster H (2006). The circadian rhythm of glucocorticoids is regulated by a gating mechanism residing in the adrenal cortical clock. Cell Metab.

[5] Nakano JJ, Shimizu K, Shimba S, Fukada Y (2016). SCOP/PHLPP1β in the basolateral amygdala regulates circadian expression of mouse anxiety-like behavior. Sci Rep.

[6] Shimizu K (2016). SCOP/PHLPP1β mediates circadian regulation of long-term recognition memory. Nat Commun.

[7] Eckel-Mahan KL (2008). Circadian oscillation of hippocampal MAPK activity and cAmp: implications for memory persistence. Nat Neurosci.

[8] Valentinuzzi VS, Menna-Barreto L, Xavier GF (2004). Effect of circadian phase on performance of rats in the Morris water maze task. J Biol Rhythms.

[9] Martin-Fairey CA, Nunez AA (2014). Circadian modulation of memory and plasticity gene products in a diurnal species. Brain Res.

[10] Takahashi Y, Sawa K, Okada T (2013). The diurnal variation of performance of the novel location recognition task in male rats. Behav Brain Res.

[11] Chaudhury D, Colwell CS (2002). Circadian modulation of learning and memory in fear-conditioned mice. Behav Brain Res.

[12] Ralph MR, Sam K, Rawashdeh OA, Cain SW, Ko CH (2013). Memory for time of day (time memory) is encoded by a circadian oscillator and is distinct from other context memories. Chronobiol Int.

[13] Koulack D (1997). Recognition memory, circadian rhythms, and sleep. Percept Mot Skills.

[14] Maury P, Queinnec Y (1992). Influence of time of 24-hour day on depth of processing in recall memory. Br J Psychol.

[15] Grabowska A, Luczywek E, Fersten E, Herman A, Szatkowska I (1994). Memory impairment in patients with stereotaxic lesions to the hippocampus and amygdala. Acta Neurobiol Exp.

[16] Tulving E, Markowitsch HJ (1998). Episodic and declarative memory: role of the hippocampus. Hippocampus.

[17] Zola-Morgan SM, Squire LR (1990). The primate hippocampal formation: evidence for a time-limited role in memory storage. Science.

[18] Ranganath C, Hsieh LT (2016). The hippocampus: a special place for time. Ann NY Acad Sci.

[19] Mingaud F (2007). The hippocampus plays a critical role at encoding discontiguous events for subsequent declarative memory expression in mice. Hippocampus.

[20] Clark RE, West AN, Zola SM, Squire LR (2001). Rats with lesions of the hippocampus are impaired on the delayed nonmatching-to-sample task. Hippocampus.

[21] Barnes CA, McNaughton BL, Goddard GV, Douglas RM, Adamec R (1977). Circadian rhythm of synaptic excitability in rat and monkey central nervous system. Science.

[22] Brognard J, Sierecki E, Gao T, Newton AC (2007). PHLPP and a second isoform, PHLPP2, differentially attenuate the amplitude of Akt signaling by regulating distinct Akt isoforms. Mol Cell.

[23] Brognard J, Newton AC (2008). PHLiPPing the switch on Akt and protein kinase C signaling. Trends Endocrinol Metab.

[24] Shimizu K, Okada M, Takano A, Nagai K (1999). SCOP, a novel gene product expressed in a circadian manner in rat suprachiasmatic nucleus. FEBS Lett.

[25] Shimizu K, Phan T, Mansuy IM, Storm DR (2007). Proteolytic degradation of SCOP in the hippocampus contributes to activation of MAP kinase and memory. Cell.

[26] Broadbent NJ, Squire LR, Clark RE (2004). Spatial memory, recognition memory, and the hippocampus. Proc Natl Acad Sci USA.

[27] Clarke JR, Cammarota M, Gruart A, Izquierdo I, Delgado-García JM (2010). Plastic modifications induced by object recognition memory processing. Proc Natl Acad Sci USA.

[28] Snider KH, Sullivan KA, Obrietan K (2018). Circadian regulation of hippocampal-dependent memory: circuits, synapses, and molecular mechanisms. Neural Plast.

[29] Kamada R (2020). Metal-dependent Ser/Thr protein phosphatase PPM family: Evolution, structures, diseases and inhibitors. Pharmacol Ther.

[30] Anbazhagan P (2008). Evolutionary analysis of PHLPP1 gene in humans and non-human primates. Bioinformation.

