# Supplementary material for: Diurnal variation in declarative memory and the involvement of SCOP in cognitive functions in nonhuman primates
Source: Mol Brain. 2023 Mar 25;16:31. doi: 10.1186/s13041-023-01022-0 (PMC10039603; doi:10.1186/s13041-023-01022-0)
Supplement: Supplementary file 1 — Additional file 1: The full image of the western blot data shown in Fig.3b in the main manuscript. The blue square is the cropped area.. [file 13041_2023_1022_MOESM1_ESM.pdf]

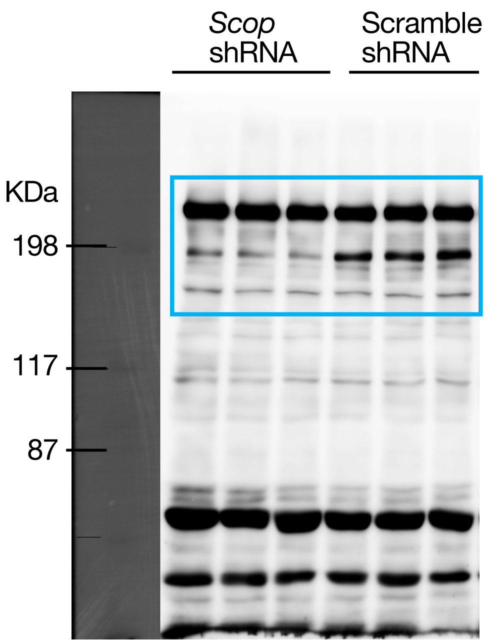

Additional file 1: The full image of the western blot data shown in Fig.3b in the main manuscript. The blue square is the cropped area.
